# Expression of Oct4A splicing variant in human bladder premalignant lesions predicts its invasiveness

**DOI:** 10.1186/1897-4287-10-S3-A9

**Published:** 2012-04-20

**Authors:** Wojciech Jóźwicki, Anna Broźyna, Wiesława Windorbska, Ewa Ziółkowska, Cezary Jochymski

**Affiliations:** 1 Department of Tumor Pathology and Pathomorphology, Oncology Centre - Prof. Franciszek Łukaszczyk Memorial Hospital, The Ludwik Rydygier Collegium Medicum, Nicolaus Copernicus University, Bydgoszcz 85-796, Poland; 2 Department of Teleradiotherapy, Oncology Centre - Prof. Franciszek Łukaszczyk Memorial Hospital, Bydgoszcz 85-796, Poland; 3 Department of Radiotherapy, Oncology Centre - Prof. Franciszek Łukaszczyk Memorial Hospital, Bydgoszcz 85-796, Poland

## 

Stem cells are immature, unspecialized cells that are able to proliferate and population self-renewal. In tumor mass, the cells with properties similar to stem cells are present. These cells, so-called cancer stem cells (CSC), are small subpopulation of cells, which can initiate the tumor and cause the recurrence of the tumor. According to published data, CSC have different level of sensitivity to chemo- and radiotherapy, dependent, i. e. on its differentiation state. It seems that complete recovery of the patients is possible only when all CSC will be eliminated. There are many of stem cell markers. In our research we analyzed the expression of OCT4A splicing variant of OCT4 (POU5F1) gene in preinvasive and malignant lesions of the bladder. OCT4A is a marker of embrional stem cells and, as transcription factor, is responsible for sustaining the cells in undifferentiated state, characteristic of the stem cell and potential to self-renewal. Our previous study suggested that its expression is observed in premalignant and malignant lesions and are consistent with results of other authors.

We examine archival, buffered formalin-fixed, paraffin-embedded tissue samples of urinary bladder specimens obtained from 113 patients. Expression of OCT4A was detected using immunohistochemistry. The intensity of OCT4A immunostaining was scored as 0 (negative), 1 (faint) or 2 (distinct) and OCT4A-positive cells were analyzed as the numbers of cells per 1000 tumor cells. Based on our results, we found the most intensive and the most extensive (measured by means of percentage of OCT4A-positive cells) expression of this marker in urothelial cells at in situ stage. In papillary tumors (pTa) and in situ cancers the percentage of OCT4A-positive cells was higher when compared to less advanced invasive cancers (pT1 and pT2), whereas the percentage of OCT4A-positive cells in pTa tumors was similar to that observed in invasive tumors at pT4 stage. The observed differences were well-defined and statistically significant.

To summarize, the highest number and the most intensive staining of OCT4A-positive cells in non-invasive lesions pTis and pTa could suggest the important role of OCT4A in initiation and promotion of invasive process. It cannot be excluded that, analysis and evaluation of the expression of this transcription factor can have predictive value in the prediction of progression of pre-invasive tumor to invasive form.

